# Carbon nanowalls as a platform for biological SERS studies

**DOI:** 10.1038/s41598-017-13087-8

**Published:** 2017-10-17

**Authors:** Pavel Dyakonov, Kirill Mironovich, Sergey Svyakhovskiy, Olga Voloshina, Sarkis Dagesyan, Andrey Panchishin, Nikolay Suetin, Victor Bagratashvili, Petr Timashev, Evgeny Shirshin, Stanislav Evlashin

**Affiliations:** 1 D. V. Skobeltsyn Institute of Nuclear Physics, M. V. Lomonosov Moscow State University, Moscow, 119991 Russia; 2 Department of Physics, M. V. Lomonosov Moscow State University, Moscow, 119991 Russia; 3 Institute of Photonic Technologies, Research center “Crystallography and Photonics”, RAS, 2 Pionerskaya st., Troitsk, Moscow, 142190 Russia; 4 Institute for Regenerative Medicine, Sechenov First Moscow State Medical University, 8-2 Trubetskaya st., Moscow, 119991 Russia; 5 Center for Design, Manufacturing and Materials, Skolkovo Institute of Science and Technology, 3 Nobel Street, Moscow, 143026 Russia

## Abstract

Herein we report about developing new type of Surface Enhanced Raman Scattering (SERS) substrates based on Au-decorated carbon nanowalls. The designed substrates possess high specific surface area and high sensitivity. Chemical stability of Au perfectly blends with electrical properties and high value of specific surface area of carbon nanowalls. Created structures were applied to detect signals of a typical molecule used for SERS substrates testing, rhodamine 6G, which exhibits electronic absorption in the visible area of spectrum, and biomacromolecules such as tryptophan, guanine, bovine serum albumin and keratin hydrolysates, whose electronic absorption is in the ultraviolet region of spectrum and lies far from the Au plasmonic resonance. The obtained signals for these compounds suggest that the developed substrate is a prominent platform for the detection of biological macromolecules. The properties of the substrate, including its morphology and Au film thickness, as well as the analyte deposition method, were optimized to achieve the optimum Raman signal enhancement. Electric field distribution in the designed structures was calculated to describe the observed dependence of SERS activity on the substrate morphology.

## Introduction

Raman spectroscopy is a powerful analytical tool, which is capable of identifying a fingerprint vibrational spectrum of analyte molecules, which are characterized by unique distributions of oscillatory frequencies. However, when it comes to measurements of analytes at low concentrations, weak cross-section of Raman scattering (~10^−29^ cm^2^) makes the signal intensity quite low and conventional Raman spectroscopy becomes ineffective. In order to detect signals from solutions with low analyte concentrations different enhancement techniques are applied. The most popular enhancement technique is Surface-Enhanced Raman Spectroscopy (SERS), which uses rough metal surfaces as substrates. Various types of SERS substrates were created during four past decades, but still there is a strong need for new methods of engineering structures able to enhance Raman signal because so far there is no low-cost technique that can combine high enhancement factor (EF), reproducibility and stability of signal all over the surface of the substrate. With a help of SERS technique, high EF (as high as ~10^14^)^1^ can be achieved, and signals from individual molecules can be measured. SERS removes sensitivity limitations of conventional Raman spectroscopy and expands its applications towards commercial needs. It is already being used for food safety improvement, trace analysis of drugs and many more^2–4^. Development of reproducible substrates with a high average EF will greatly improve SERS involvement in practical applications. SERS is a complex analytical tool, whose performance depends on multiple factors. One of the them (and probably the most important) is a substrate choice. So, which substrates should be considered as good ones? First of all, substrate should be made with a use of “correct” materials: metals such as gold and silver are most popular, since their films have appropriate optical and electronic properties. However, more materials are discovered to have plasmonic properties lately and thus potentially can be used for enhancing Raman signals^5^. Secondly, substrates need to contain objects of subwavelength size on their surfaces. Thirdly substrates should possess high specific surface area to provide higher number of adsorbed molecules per the same surface area unit^6,7^. On the other hand, the major challenge for developing substrates is the reproducibility of measurement results. Highest known EFs are obtained for signals of molecules adsorbed on the apex of the metal needle (so-called Tip-Enhanced Raman Spectroscopy)^8^, but these measurements require significant effort for pretreatment and signal aquisition. For express analysis it is crucial to be able to obtain analyte signal from as many spots of the substrate as possible. Most of the commercial applications require not the highest enhancement factor, but reproducibility and uniform distribution of the hot spots all over the substrate surface. Such qualities provide the best conditions for fast and reliable measurements. In this work we have chosen the strategy of decoration of films of high specific surface area with Au particles, to obtain uniform and dense distribution of hot spots all over the substrate. As a substrate forming surface we have chosen Carbon NanoWalls (CNWs) - a dense array of vertically oriented graphitic structures (“walls”) separated with spacers of tens-hundreds of nanometers. Each “wall” consist of several graphene layers, and the number of graphene layers can vary from a single layer to tens of layers. CNWs exhibit high surface area of 1000 m^2^/g^9^ due to nanostructures on its surface. These properties are crucial for production of substrates with a high average enhancement. Also both CNWs and golden particles appear to be biocompatible, that is essential for good SERS sensing of biological objects. Recently, involvement of various carbon materials such as raw and modified graphene^10^, carbon nanotubes^11^ and various modifications^12^, 3D graphene foam^13^ and etc. in production of SERS substrates increased greatly. For instance, in paper^14^ it was shown that raw graphene can enhance Raman signal and selectivity of graphene enhanced Raman scattering was studied. CNW substrates already were also used for SERS measurements by a few groups. In work^15^ SERS behavior of Ag nanoparticles and thin films on CNWs was studied. The work^15^ studied SERS performance of electrochemically deposited Ag nanoparticles on CNWs. In aforementioned works Ag shows excellent SERS activity but due to the chemical instability and tendency to degrade by exposure to air it is hardly suitabe for quantitative SERS studies. At the same time, chemical stability of Au makes SERS signal provided by Au more uniform and reproducible. Also chemical stability of Au allows to perform experiments on substrates exposed on air without any considerable degradation. In this work, we demonstrate new type of homogeneous SERS-substrates with a high sensitivity, density of hot spots and reproducibility of signal all over the substrate surface. Substrates were prepared by magnetron sputtering of Au films on Carbon NanoWalls (CNWs) and were applied to investigate the limits of detection of Rhodamine 6 g (R6G), Bovine Serum Albumin (BSA), tryptophan, keratin and guanine. We show that CNW substrates could be used to detect low concentrations of organic molecules (up to 10 nM). Furthermore, to provide deeper analysis of SERS behaviour of Au@CNW substrates, we carried out theoretical calculations of the electric field distribution in the designed system.

## Methods

### Sample preparation

We fabricated substrates via a simple two-step process: CNW synthesis and CNW coating. For both stages we had control over experimental conditions and, thus allowing us to have reproducibility of morphologies of the substrates as well as regimes of Au-particle decorations, which in turn means that substrates prepared at equal conditions would provide roughly equivalent average EF. The CNW films were grown on silicon substrates in the plasma of direct current (DC) glow discharge in a mixture of methane and hydrogen^16,17^. Three types of samples were examined. Standard CNW film was grown in a standard regime corresponded to discharge current and voltage of 0.9 A and 670 V, respectively, a pressure of the working gas mixture of 100 Torr, H 2 and CH 4 flow rates of 166,7 and 16,7 sccm, respectively and the substrate temperature of 1000 °C with the growth duration of 30 minutes. CNWs with increased height (further referred as BIG CNWs) were grown when growth duration was prolonged to 60 minutes while other synthesis conditions remained unchanged. Finally CNWs with unique hierarchical morphology characterized by presence of small secondary nanowalls on the side surface of the primary nanowalls (further referred as SN CNWs) were grown in a two step process described elsewhere [Tailoring of the carbon nanowall microstructure by sharp variation of plasma radical composition]. Briefly, during the first 20 minutes growth parameters were set as follows: discharge current and voltage of 0.7 A and 690 V, respectively, a pressure of 150 Torr and substrate temperature of 1000 °C. Then discharge current was sharply increased to 0.9 A for 5 minutes with a corresponding increase in temperature to 1080 °C with other parameters being constant. Decoration of the CNWs was carried out by DC magnetron sputtering. The sputtering of aurum target of high purity (99,999% purity) was conducted in chamber of argon at pressure of 50 mTorr. Power density per unit of substrate surface was 5.5 W/cm^2^. Sputtering rate was 0.8 nm/sec and distance between substrate and target was 5 cm. Sputtering was performed for different times in range from 50 to 250 seconds.

### Preparation of test analyte

Solutions of several analytes were used to investigate the quality and sensitivity of SERS substrates. First, rhodamine 6 G dye (Sigma-Aldrich, product number 252433) was used. Isopropanol R6G solutions were prepared with concentrations in the 10^−9^–10^−5^ M range. Next, we used L-tryptophan (Sigma-Aldrich, product number T8941) as a model organic compound with a low molecular weight, whose electronic resonance (280 nm) is far from the plasmonic resonance of Au films, to test the applicability of the developed SERS substrates for detection of biologically relevant molecules. Finally, isopropanol solutions of Bovine Serum Albumin (BSA) (MP Biomedicals, product number 02152401) and keratin hydrolysates were chosen as a test biomacromolecular objects. Isopropanol solution of tryptophan was prepared of 99% Sigma-Aldrich L-Tryptophan (Product number T8941). Keratin water solution was prepared from a human nail as it was described in^18^. To apply the solutions to the substrate, the 50 *μ*l Discovery micropipette was used.10 *μ*l of solution was applied to all substrates. Surface area of substrates was roughly 0.25 cm^2^. Prepared substrates are sensitive to the choice of solvent, see Fig. S1.

### Sample analysis

The morphology of CNW films was studied using optical (Carl Zeiss Axio Vision) and scanning electron microscopy (SEM) (Carl Zeiss Supra 40 system). SERS microscopy was performed using 3D Scanning Laser Confocal Raman Microscope Nanofinder-S (Solinsruments, Belorus) using the 635 nm excitation wavelength and 40x objective, and the laser power at the surface of the sample did not exceed 10 mW.

### Numerical calculation of electromagnetic field distribution

The calculation was performed by two-dimensional finite-difference time-domain (FDTD) method using MEEP code^19^. The calculation region was 2 × 2 *μ*m^2^, spatial resolution was 0.5 nm per grid unit. Incident light was a plane wave with a constant wavelength of 630 nm. The duration of simulation was of 25 periods of incident light wave so the electromagnetic field distribution had reached stationary state. The structure of gold-plated carbon nanowalls was provided as follows: single carbon nanowall was modelled as solid block with length of 1 *μ*m and thickness of 10 nm, the dielectric permittivity at 630 nm was *ε* = 5.4904 + 9.0137i the same as bulk graphite^20^. Gold layers were modelled as randomly placed gold spheres with diameter of 30 nm which is close to average roughness of the surface. Gold spheres were overlapped to create bulk gold without voids; overlapping factor was of 20. The dielectric permittivity of gold was modelled using the Drude dispersive model. Material constants for Au were taken from^21^.

## Results

To characterize morphologies of raw CNWs we introduced such parameters as average “wall” height and thickness and number of CNWs per unit of surface area (Table 1). All the data in the Table 1 was obtained from average measurements for 10 of SEM images for each morphology. The parameter “Film thickness” represents the distance from the surface of the substrates to the tip of CNWs. To obtain mean CNW surface density SEM images of 64 square micrometer regions were considered. Mean number of “walls” on these images was divided by surface area of region in order to achieve mean surface density. Mean CNW size was obtained by measuring the length of on CNWs on multiple SEM images and then calculating its mean value.

**Table 1:** Physical parameters of different CNWs without coatings.

	Standard CNW	SNCNW	Big CNW
Film thickness, *μ*m	2.5	3.8	5.2
Mean CNW surface density, *μ*m^−2^	6.7	15.2	9.8
Mean CNW size, nm	595	309	446

**Figure 1:**
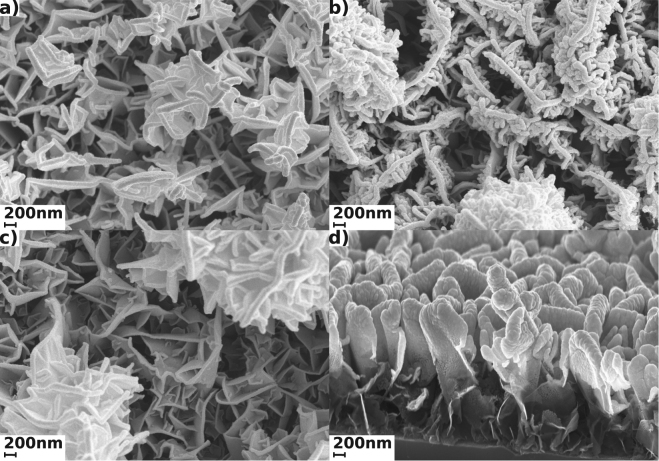
SEM images of gold coated CNWs for 100 seconds of Au-sputtering. Different morphologies are presented: (**a**) shows SEM image of Standard CNW, (**b**) is SEM for SNCNW morphology, (**c**) is SEM image of BIG CNWs and (**d**) shows inclined SEM of Standard CNWs.

Figure 1 presents SEM images of CNW@Au composite substrates. Images show homogeneous distribution of sputtered Au particles on the edges of the “walls”. Such distribution is belived to provide uniform SERS signal of analyte all over the substrate, as uniformity of signal all over the substrate is required for analysis of samples with low concentrations. Uniformity allows to quantitatively understand concentration of sample under investigation and to simplify the process of measurement. Also from the SEM images average thickness of the “walls” was estimated for each morphology: for samples after 100 seconds of magnetron sputtering of Au-films the average thickness of decorated walls was 82 nm for SN, 57 nm for Big and 87 nm for Standard.

**Figure 2:**
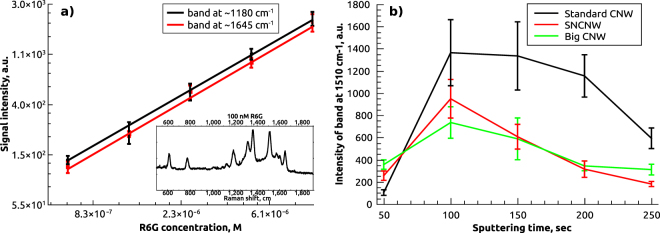
Results of R6G measurement on CNW@Au, (**a**) shows calibration curves for two different R6G bands and average spectrum of 100 nM R6G solution (in the inset), (**b**) illustrates the dependence of R6G signal intensity on the thickness of sputtered Au layer for three different morphologies.

To demonstrate applicability of the designed SERS substrates for quantitative measurements, we measured calibration curves for R6G. All data was collected from 10 different spots on the substrates surface. Collected data was averaged, fitted linearly and plotted with error bars in a double logarithmic scale. Linear dependence in the log-log scale between signal intensity for two characteristic R6G bands and R6G concentration was observed for the concentration range 10^−5^–10^−7^ M (Fig. 2a). In order to estimate experimental detection limit we performed studies using R6G solutions of various concentrations. The lowest concentration which provided signal with intensity above detection limit, defined according to^22^, was 1 nM. To get a deeper insight into the mechanism of SERS-activity of CNW@Au composite substrates we studied the dependence of the average signal intensity on thickness of sputtered layer of Au films. R6G was used as a test analyte. Similar R6G solution was used to obtain signals for samples of different morphologies prepared with various sputtering times. Figure 2b shows the result of investigation for R6G Raman spectrum band at 1510 cm^−1^. As a result of this analysis “optimum” thickness for each morphology had been determined. Sputtering time was changed with a 50 s interval, and for each sample we collected signals of R6G from random spots on the substrate surface. As one can see from Fig. 2b, the best average enhancement was obtained for the sputtering time of 100 seconds for each morphology. More importantly standard CNWs after 100 seconds of sputtering showed the best EF (Fig. 2b)). This allowed us to use only Standard samples with 100 seconds Au-coatings for further studies. Of two substrates with different surface area under other equal conditions, the one with higher specific surface area should provide stronger signal, due to the fact that higher specific surface area provides larger number of adsorbed analyte molecules. As reader can see from Table 1 SNCNW possess the highest mean wall surface density. However, experimental study shows that Standard CNWs demonstrate the strongest enhancement among other morphologies (see Fig. 2b)). The possible explanation could be that SNCNW morphology provides the highest electromagnetic absorption in visible part of spectrum^23^, thus reducing the efficiency of plasmon excitation. Also thickness and distribution of Au layer on CNWs should be taken into consideration and exact explanation for this phenomenon is still to be found.

**Figure 3:**
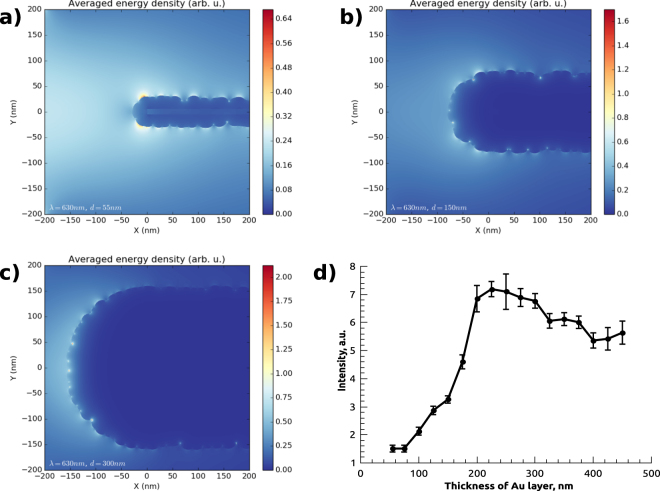
Results of calculation of plasmon activity for Au particles on carbon “wall”, figures (**a**–**c**) show average energy density distributions for Au-coating of 55 nm, 150 nm and 300 nm thickness, correspondingly. (**d**) shows the dependence of calculated enhancement intensity on thickness of Au layer.

In order to further characterize SERS activity of CNW@Au substrate, we carried out theoretical simulation of plasmon behavior on the surface of Au-decorated CNW substrates. Results of the numerical calculation are shown in Fig. 3 and illustrate plasmon activity of single Au-coated carbon “wall” of different thickness. General outline of theoretical dependence is in a satisfactory agreement with experimental data shown in Fig. 2b. However, the performed calculation only shows results for plasmon activity of a single “wall” and does not take interaction between nearby walls into consideration.

**Figure 4:**
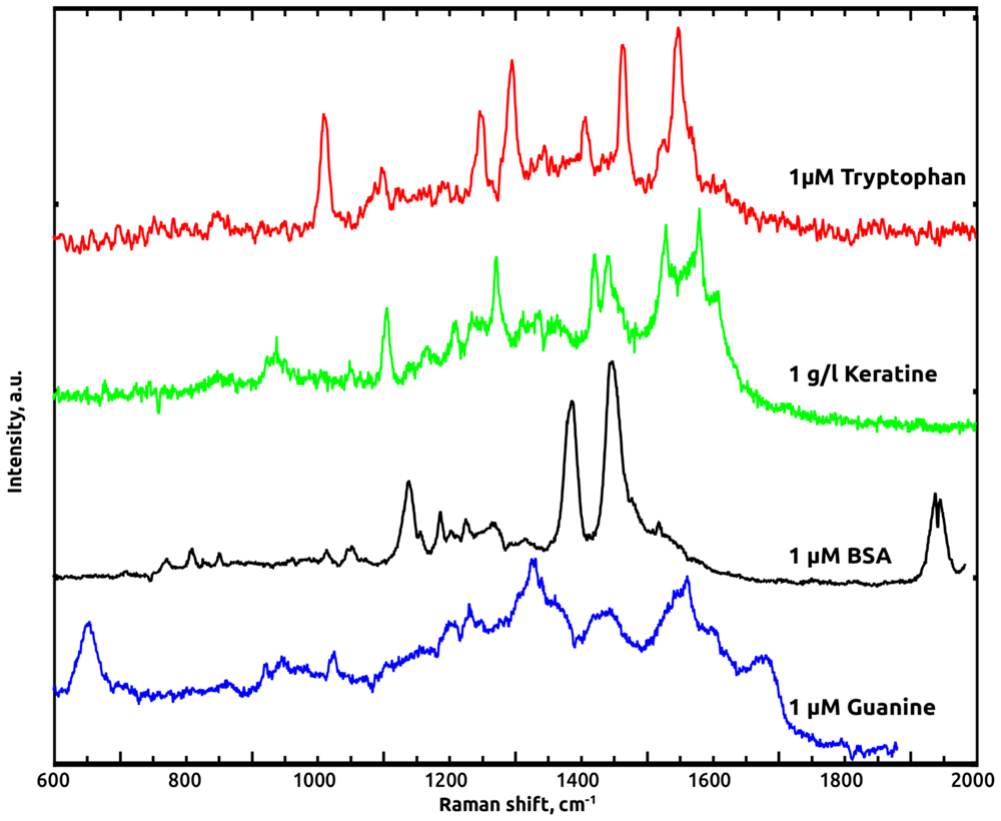
Raman spectra of biological macromolecules, from top to bottom: 1 *μ*M tryptophan (red curve), 1 g/l keratin (green curve), 1 *μ*M BSA (black curve) and 1 *μ*M guanine (blue curve).

Measurements of R6G signals is an essential part in the study of SERS-active substrates, however, R6G is mainly as a test probe, while other molecules, lacking absorption in the visible range of spectrum, are of practical importance. Hence, to verify the potential of CNW@Au substrates for biomedical applications we studied SERS-activity for biological macromolecules, whose electronic absorption values located far from Au plasmonic resonance excitation. Four types of molecules were used for testing of the prepared substrates: amino acid Tryptophan, nucleobase guanine, globular protein BSA and hydrolysates of keratin, the major protein of the outer layer of skin^18^. These molecules differ by size and molecular weight, while their absorption properties are rather similar - all the molecules demonstrate electronic absorption in the 250–280 nm range. Isopropanol solutions of Tryptophan, BSA, guanine and water solution of keratin were applied to the substrates, dried in air and then Raman spectra were measured. In this case, appropriate signals were obtained for 1 *μ*M solutions of tryptophan, BSA and 1 g/l keratin solution. Figure 4 shows the obtained spectra for the aforementioned molecules.

To study homogeneity and reproducibility of SERS signal, we used the mapping method for measuring spatial distribution of the 100 nM R6G solution signal (Fig. S2). Mapping was performed for R6G band at ~1510 cm^−1^ and showed average hot spot density of about 10 hot spots per *μ*m^2^. Also mapping at 1350 cm^−1^ was performed for BSA solutions of 1 *μ*M to study uniformity of signal of biological objects. The average hot spot density for BSA was also roughly ~10 hot spots per *μ*m^2^. The fact that hot spot densities for BSA and R6G were roughly equal verifies the suggestion that Au@CNW substrates are efficient for both standard SERS analyte (R6G) and proteins (BSA). Noteworthy is the fact that sometimes the resulting spectrum is interfered by the Raman signal of CNWs. This problem can be solved by subtraction of the baseline Raman spectrum from resulting signal^16^.

## Conclusion

In this work we demonstrate new type of SERS substrates based on Au-coated CNWs. These substrates posses dense and homogeneous distribution of hot spots, high EF and do not tend to degrade with time due to exposure to air. Dependencies of the EF on the Au layer thickness and CNWs morphology were studied. The developed substrates were applied for investigation of signals from both classical Raman analyte R6G and macromolecules such as keratin, BSA, tryptophan and guanine, which do not have electronic absorption in the vicinity of Au plasmonic resonance. Minimum detectable concentrations were 1 nM for R6G, 1 *μ*M for BSA, tryptophan and guanine. Also, to describe obtained substrates theoretically we performed calculation of plasmonic activity of Au particles on CNWs. Good agreement between the results of numerical calculation of electromagnetic field intensity distribution and experimental data was achieved.

## Electronic supplementary material

Supplementary information
