# Publisher Correction: Carbon nanowalls as a platform for biological SERS studies

**DOI:** 10.1038/s41598-018-21794-z

**Published:** 2018-03-06

**Authors:** Pavel Dyakonov, Kirill Mironovich, Sergey Svyakhovskiy, Olga Voloshina, Sarkis Dagesyan, Andrey Panchishin, Nikolay Suetin, Victor Bagratashvili, Petr Timashev, Evgeny Shirshin, Stanislav Evlashin

**Affiliations:** 1 D. V. Skobeltsyn Institute of Nuclear Physics, M. V. Lomonosov Moscow State University, Moscow, 119991 Russia; 2 Department of Physics, M. V. Lomonosov Moscow State University, Moscow, 119991 Russia; 3 Institute of Photonic Technologies, Research center “Crystallography and Photonics”, RAS, 2 Pionerskaya st., Troitsk, Moscow, 142190 Russia; 4 Institute for Regenerative Medicine, Sechenov First Moscow State Medical University, 8-2 Trubetskaya st., Moscow, 119991 Russia; 5 Center for Design, Manufacturing and Materials, Skolkovo Institute of Science and Technology, 3 Nobel Street, Moscow, 143026 Russia

Correction to: *Scientific Reports* 10.1038/s41598-017-13087-8, published online 17 October 2017

In the original version of this Article, there were errors in Affiliation 4 which was incorrectly listed as:

“Institute for Regenerative Medicine, First Moscow State Medical University, 8-2 Trubetskaya st., Moscow, 119991, Russia”.

The correct affiliation is listed below:

“Institute for Regenerative Medicine, Sechenov First Moscow State Medical University, 8-2 Trubetskaya st., Moscow, 119991, Russia”.

These errors have now been corrected in the PDF and HTML versions of the Article.

