# Supplementary material for: Carbon nanowalls as a platform for biological SERS studies
Source: Sci Rep. 2017 Oct 17;7:13352. doi: 10.1038/s41598-017-13087-8 (PMC5645332; doi:10.1038/s41598-017-13087-8)
Supplement: Supplementary file 1 — Supplementary information [file 41598_2017_13087_MOESM1_ESM.pdf]

Supplementary material for manuscript:

# Carbon nanowalls as a platform for biological SERS studies.

Pavel Dyakonov[1✉], Kirill Mironovich[1], Sergey Svyakhovskiy[2], Olga Voloshina[2], Sarkis Dagesyan[2], Andrey Panchishin[2], Nikolay Suetin[1], Victor Bagratashvili[3], Petr Timashev[3,4], Evgeny Shirshin[2], Stanislav Evlashin[5]

[1] D. V. Skobeltsyn Institute of Nuclear Physics, M. V. Lomonosov Moscow State University, Moscow, 119991, Russia

[2] Department of Physics, M. V. Lomonosov Moscow State University, Moscow, 119991, Russia

[3] Institute of Photonic Technologies, Research center "Crystallography and Photonics", RAS 2 Pionerskaya st., Troitsk, Moscow, 142190, Russia

[4] Institute for Regenerative Medicine, , Sechenov First Moscow State Medical University, 8-2 Trubetskaya st., Moscow, 119991, Russia

[5] Center for Design, Manufacturing and Materials, Skolkovo Institute of Science and Technology, Center for Design, Manufacturing and Materials, 3 Nobel Street, Moscow, 143026, Russia

Email: djjakonov.pavel@physics.msu.ru

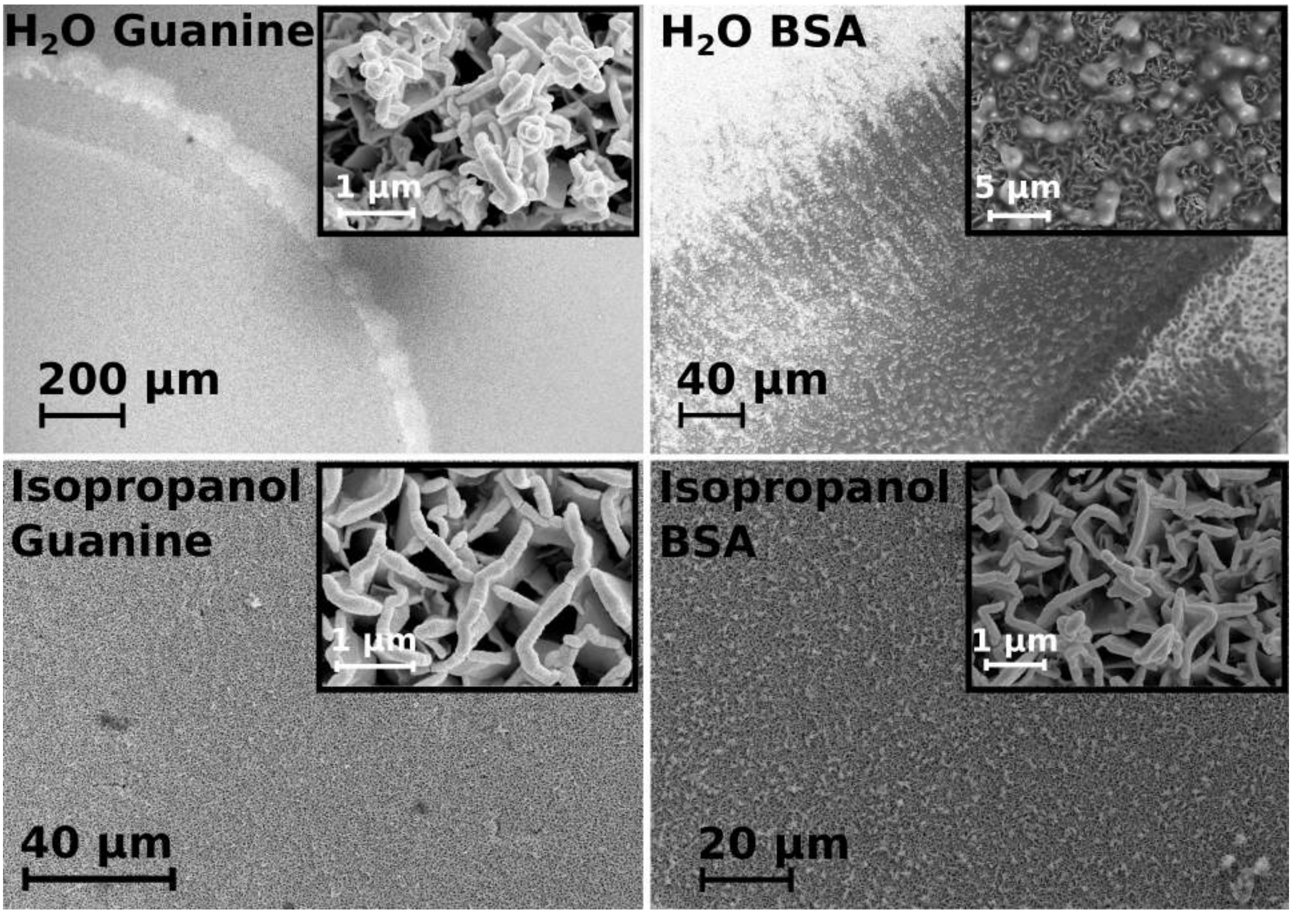


Figure S1. SEM images of drying process of analytes in different solvents (water and isopropanol).

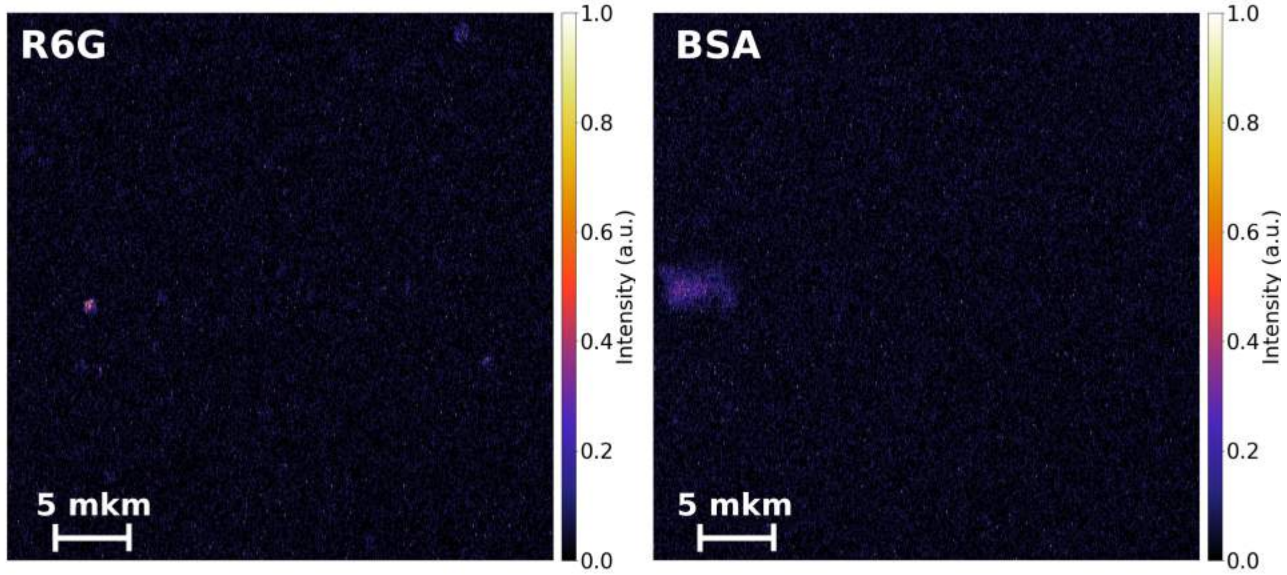


Figure S2. Spectral maps for 0.1 mkM of R6G and BSA solutions.

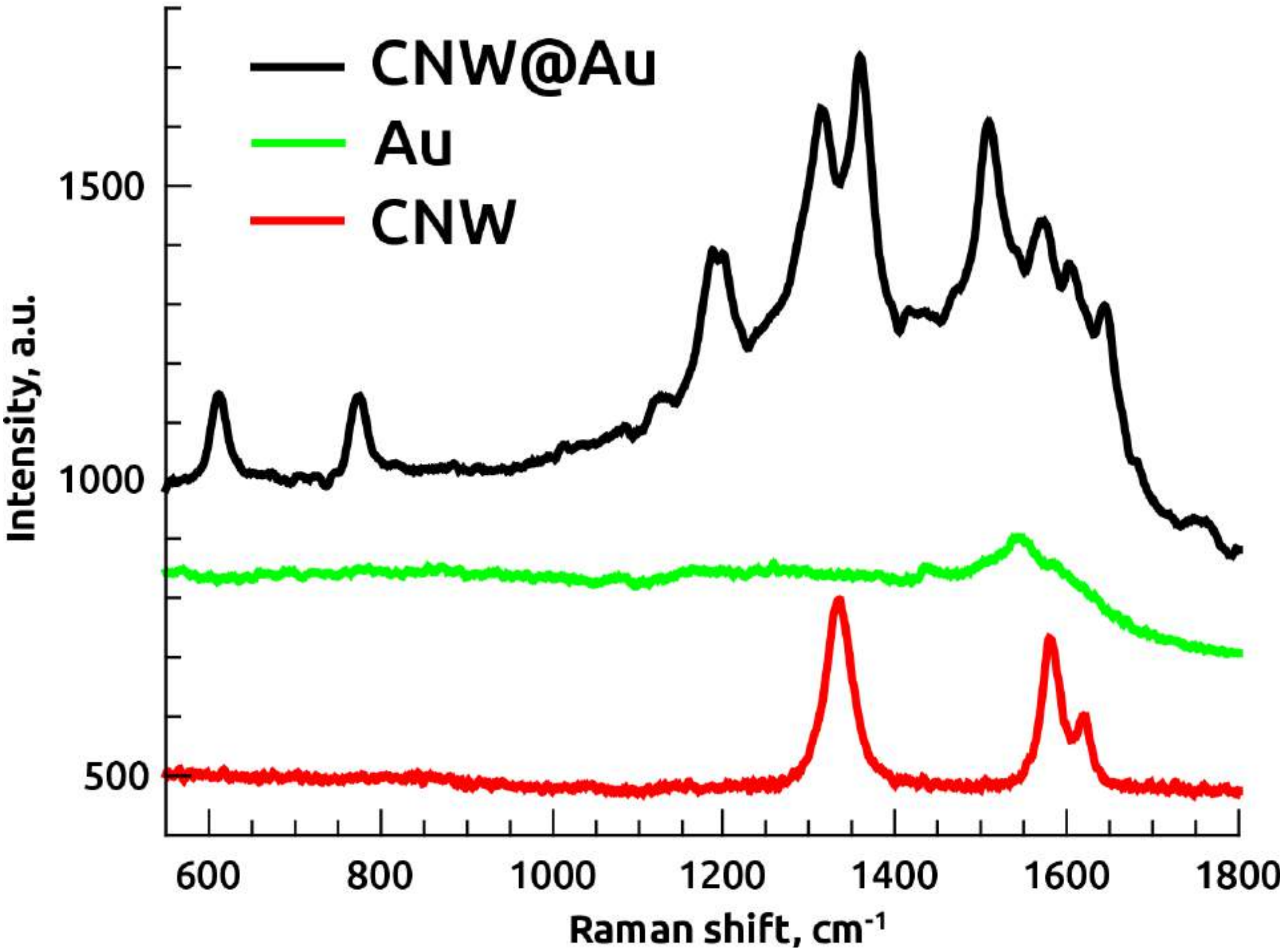


Figure S3. 1 mkM R6G solution applied to substrates of raw CNWs (red curve), plane Au (green curve) and CNW@Au.
